# Differential pathways from arts and creative activities to student learning and wellbeing: the roles of belonging, self-efficacy, and self-regulation

**DOI:** 10.3389/fpsyg.2026.1905846

**Published:** 2026-09-17

**Authors:** Song Dong, Boda Wang, Jianyun Wei

**Affiliations:** 1 Department of Global Convergence, Kangwon National University, Chuncheon-si, Gangwon-do, Republic of Korea; 2 Department of Composition, Harbin Conservatory of Music, Harbin, Heilongjiang, China

**Keywords:** arts and creative activities, creative thinking, PISA 2022, school belonging, self-efficacy, self-regulation, student wellbeing

## Abstract

**Background:**

Participation in arts and broader creative activities is associated with students’ learning, emotional development, and wellbeing, but the psychological processes accompanying these associations remain insufficiently understood. Because all focal variables were observed in the same PISA 2022 cycle, the present study treats the proposed pathways as cross-sectional indirect-association patterns rather than as established temporal or causal mechanisms.

**Objective:**

This study examines whether school belonging, self-regulation, self-efficacy, and creative thinking show different cross-sectional indirect-association patterns linking a broad measure of arts-and-creative-activities participation with reading performance and subjective wellbeing. The comparison concerns outcome-specific association patterns and does not assume that participation temporally precedes the proposed intervening variables.

**Methods:**

A quantitative cross-sectional secondary-data design was employed using the Programme for International Student Assessment (PISA) 2022 dataset. The analytical sample comprised 441,183 15-year-old students from 64 countries and economies that administered the creative-thinking assessment and provided linkable student-questionnaire data. Participation was represented by a bounded breadth index with an observed range of 0–8 (primary weighted M = 2.34, SD = 1.82). Distributional checking in the analysis-ready PISA subset confirmed concentration toward lower breadth values (median = 2, IQR = 1–4, skewness = 0.83). Structural equation modeling estimated pooled direct and joint indirect associations through school belonging, self-regulation, self-efficacy, and creative thinking. The models are interpreted as survey-weighted pooled associations; no country-specific latent-mean or structural-path comparisons are made.

**Results:**

Participation in arts and creative activities was positively associated with reading performance (total association β = 0.18, *p* < 0.001) and life satisfaction (total association β = 0.24, *p* < 0.001). In the full parallel models, the joint indirect association through the four prespecified constructs was β = 0.11 for reading performance and β = 0.15 for life satisfaction. A mutual-adjustment sensitivity analysis showed that the standalone ordering was not stable for reading once the four intervening constructs were considered simultaneously. The main interpretation therefore rests on the aggregate direct, joint indirect, and total associations rather than on a strongest-mediator claim.

**Conclusion:**

The pooled models identify positive direct and joint indirect associations between a heterogeneous arts-and-creative-activities breadth measure and both outcomes. The cross-sectional design does not establish temporal or causal mediation, and the analysis does not make strongest-mediator claims. The multi-country estimates are interpreted as survey-weighted pooled averages rather than evidence that structural relations are identical in every country. The findings apply to the broad participation construct and do not demonstrate effects of formal arts education or any single art form.

## 1 Introduction

Recent research has increasingly linked arts and broader creative participation with cognitive, social, emotional, and wellbeing-related outcomes in young people (Fancourt and Finn, 2019; Patalay and Fitzsimons, 2018; OECD, 2024). This literature has encouraged a broader view of student development beyond achievement in core academic subjects. At the same time, evidence varies by activity, design, population, and outcome, and observational associations should not be interpreted as proof that participation itself produces later change. This distinction is particularly important for large international assessments, where multiple constructs are measured within the same assessment cycle.

In 2022, PISA formally assessed creative thinking alongside its established academic domains (OECD, 2024). Barbot and Kaufman (2025) are cited for their discussion of the PISA 2022 creative-thinking assessment; their analytical sample is not used to define the present study. Here, *N* = 441,183 follows the study-specific inclusion rule: students from the 64 countries and economies that administered the creative-thinking assessment and had linkable student-questionnaire records meeting the analysis requirements. Longitudinal studies outside PISA have also linked sustained arts, culture, and entertainment participation with later wellbeing (Thornton et al., 2024), while performing-arts research has reported positive mental-health associations (Block et al., 2022). These findings motivate examination of psychological correlates without establishing the temporal ordering of the variables used here.

School belonging, self-regulation, and self-efficacy are theoretically relevant candidate correlates of the participation-outcome relationship. School belonging reflects students’ perceived acceptance and connection within school (Allen et al., 2021) and is associated with wellbeing and academic outcomes (Gopalan and Brady, 2020; Korpershoek et al., 2020; Štremfel et al., 2024). Self-regulation concerns the planning, monitoring, and evaluation of learning (Panadero et al., 2018) and is associated with educational performance (Tuero et al., 2022; Morosanova et al., 2023). Self-efficacy refers to beliefs about one’s capacity to carry out goal-directed actions (Bandura, 1997) and has been associated with academic success and psychological wellbeing (Schunk and DiBenedetto, 2020; Luo et al., 2023). In the present cross-sectional study, these constructs are treated as theoretically motivated intervening associations, not as temporally established mediators.

Although previous research has linked arts and creative participation with academic and wellbeing outcomes, less is known about whether correlated intervening associations differ across outcome domains when several constructs are considered in one model. PISA 2022 provides an opportunity to examine school belonging, self-regulation, self-efficacy, and creative thinking within a large international dataset. Throughout this study, “indirect association” denotes a statistical decomposition under a prespecified cross-sectional model; it does not establish that participation occurred first, that an intervening construct changed next, or that the outcome changed last. Consistent with the unchanged title, “differential pathways” is used only as descriptive shorthand for differences in modeled association patterns across constructs and outcomes.

In this manuscript, “arts and creative activities” denotes the heterogeneous set assessed in PISA 2022 and is not used synonymously with formal arts education. Music, visual arts, creative writing, and drama or theater are conventional arts-related activities, whereas debate, publications, science club, and computer programming are broader creativity-related activities. The study therefore examines breadth of participation in the complete PISA activity set rather than participation in a coherent formal arts curriculum. Figure 1 presents the prespecified cross-sectional indirect-association model; its arrows denote the statistical specification and not temporal or causal ordering.

**FIGURE 1:**
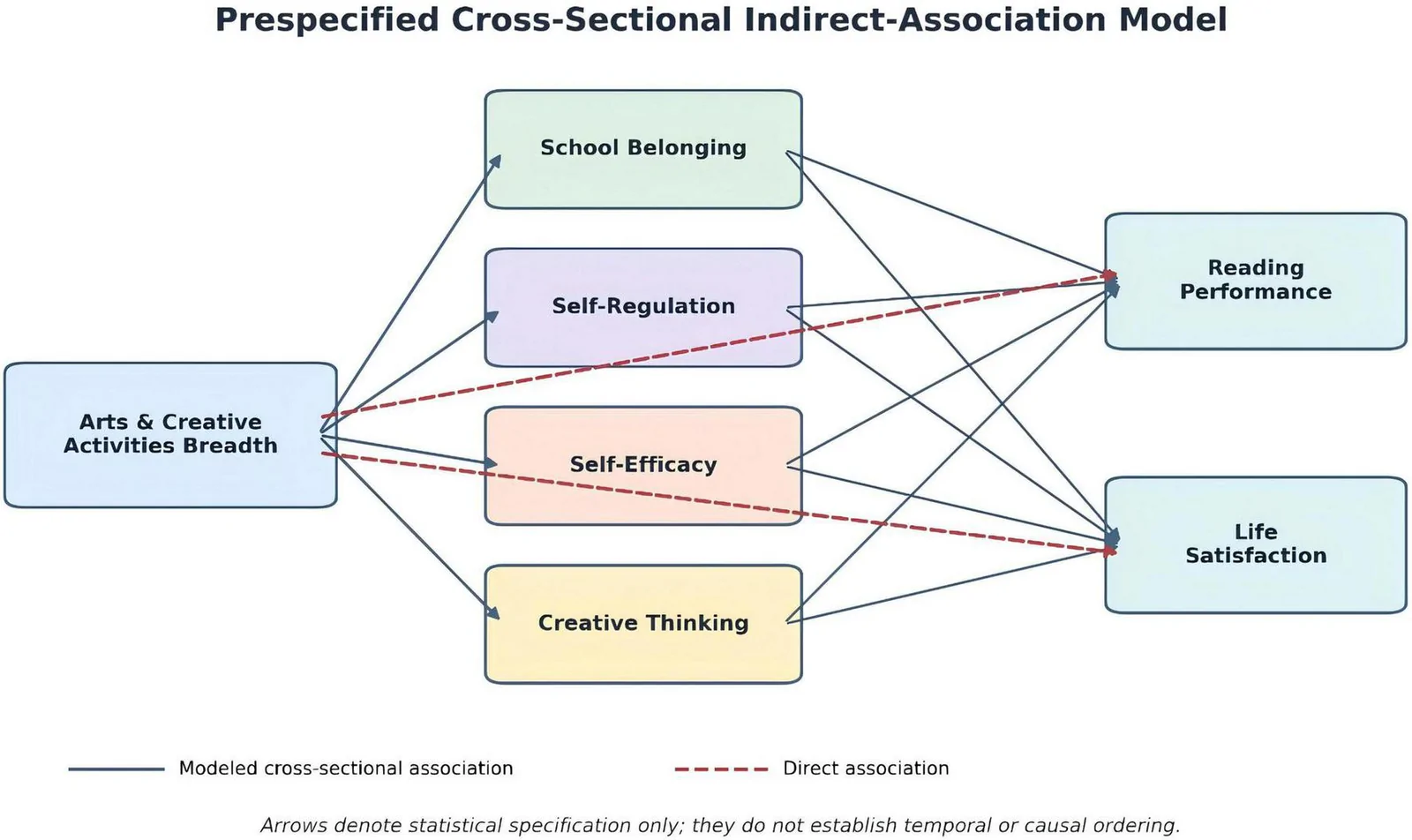
Prespecified cross-sectional indirect-association model. Arrows denote the statistical specification of the model and do not establish temporal or causal ordering.

## 2 Literature review

### 2.1 Arts education and student outcomes

Research on arts participation and student outcomes has expanded substantially, but the evidence base varies in design, population, activity definition, and causal strength. Reviews have reported positive associations between arts engagement and academic, social, and wellbeing outcomes (Winner et al., 2013; Fancourt and Finn, 2019), while experimental and quasi-experimental studies provide stronger evidence for selected school-based interventions. Schneider and Rohmann (2021), for example, reviewed experimental and quasi-experimental arts-education studies across cognitive, social, and emotional outcomes. Other work has reported associations between arts-rich participation and academic performance (Catterall et al., 2012; Liu, 2023) and between arts education, self-efficacy, and emotional development (Su and Adenan, 2025). These literatures motivate the present model, but their findings should not be generalized automatically to the heterogeneous PISA participation index used here.

The study by Block et al. (2022) also reported that frequent performing-arts participation was associated with positive mental health in young adults, while McCrary et al. (2021) synthesized evidence on the health relevance of music and dance participation. Critical reviews nevertheless identify important limitations in the literature, including small or homogeneous samples, selective outcome reporting, short follow-up periods, and uncertainty about the psychological processes that accompany participation (Hugh-Jones et al., 2025). The present study addresses some of these limitations by using a large, heterogeneous PISA 2022 analytical sample and a common set of psychological constructs. It does not, however, assume that pooling 64 countries establishes cross-country measurement equivalence or invariant structural associations.

#### 2.1.1 Music participation within the broader creative-activity measure

Music is one of the conventional arts-related activities included in the eight-activity exposure and is the activity most directly aligned with the focus of the special issue. Music participation may involve repeated practice, attentional control, performance feedback, persistence, and mastery experiences. Ensemble participation may additionally provide opportunities for coordination, peer interaction, recognition, and school belonging. These processes overlap with the constructs examined in the present study, particularly self-regulation, self-efficacy, and belonging (Block et al., 2022; James et al., 2024; Thornton et al., 2024).

Nevertheless, the primary exposure is not music-specific. Music constitutes one component of a heterogeneous activity measure that also contains visual arts, creative writing, drama, debate, publications, science club, and computer programming. Consequently, the composite-model estimates must not be interpreted as evidence of a music-specific association. Music is therefore treated as a theoretically important component of the broader exposure. Because the model does not isolate a music coefficient, the study does not attribute the composite association to music and does not claim that music differs statistically from the other activities.

### 2.2 Psychological constructs: belonging, self-regulation, and self-efficacy

School belonging is characterized as feeling valued, accepted and connected to the school environment (Allen et al., 2021). The significance of belonging has repeatedly been shown to be a major factor that impacts academic and wellbeing outcomes. Allen et al. (2021) conducted a systematic review and found that the link between school belonging and academic motivation, mental health, and decreased dropout rates are well supported by evidence from more than 200 studies. Korpershoek et al. (2020) synthesized 82 correlational studies and found consistent relationships between school belonging and motivational, social-emotional, behavioral, and academic outcomes in secondary education. A recent systematic review following PRISMA 2020 identified individual, relational, and institutional conditions associated with students’ sense of school belonging across a diverse literature (Štremfel et al., 2024). Collaborative, performative, and communal arts activities have been theorized to be associated with stronger school belonging, which may in turn be related to wellbeing.

Self-regulation is defined as the ability to plan, monitor, and adjust one’s learning strategies and behaviors (Panadero et al., 2018). It has been consistently identified as one of the most powerful predictors of academic achievement across educational levels and cultural contexts (Dent and Koenka, 2016). A school-based experimental study by Tuero et al. (2022) demonstrated that training in self-regulation learning strategies produced significant short- and long-term gains in academic performance across multiple subject areas, including mathematics, sciences, and language. Morosanova et al. (2023) found, in a 3-year longitudinal study of 500 young adolescents, that conscious self-regulation functioned as a meta-resource for academic performance and psychological wellbeing during developmental transitions, with higher self-regulation consistently predicting more positive outcomes. A related empirical study by Morosanova et al. (2022) further demonstrated that conscious self-regulation, alongside motivational and personality factors, was a significant linear predictor of academic performance in school populations. The results indicate that arts programs that incorporate a systematic approach to setting goals, reflecting on experiences, and developing skills may positively impact students’ self-regulation skills, leading to benefits for students’ academic outcomes. According to Bandura (1997), self-efficacy is the person’s belief in his or her ability to engage in the behaviors required to achieve specific results and has been linked to academic performance and psychological wellbeing. Schunk and DiBenedetto (2020) found that self-efficacy beliefs were a motivational factor that accounted for academic achievement even after controlling for previous academic performance and general cognitive ability, suggesting self-efficacy’s incremental validity as a motivational construct. Based on a recent synthesis by Luo et al. (2023), academic self-efficacy could be seen as a strong predictor of learning engagement among Chinese college students, which further mediated the relationship between academic self-efficacy and academic achievement.

Importantly, Jin and Ye (2022) concluded that the positive and significant relationship between fine arts education and psychological wellbeing was significantly mediated by self-efficacy and creativity among undergraduate students. Su and Adenan (2025) built on these findings by showing that self-efficacy lies on the path from visual arts education to academic performance and emotional regulation skills, and thus provides a mechanism for how arts engagement can lead to broader student outcomes. Basileo et al. (2024) found that self-efficacy played a major role in the relationships among perceived support for basic psychological needs, motivation, and academic achievement.

### 2.3 Creative thinking as an additional cognitive correlate

Creative thinking is conceptually related to, but not identical with, reading literacy. PISA defines creative thinking as the capacity to generate diverse and original ideas and to evaluate and improve ideas across written, visual, social-problem-solving, and scientific-problem-solving contexts, whereas reading literacy focuses on understanding, using, evaluating, reflecting on, and engaging with texts. Participation in arts and broader creative activities may be associated with repeated ideation, experimentation, refinement, and evaluation, which provides a theoretical reason to examine creative thinking as a cognitive correlate of the participation-reading association (OECD, 2024). The substantial empirical correlation between creative thinking and reading reported in this study (*r* = 0.52) also means that the two constructs share meaningful variance. Creative thinking could therefore plausibly be treated as a parallel cognitive outcome in an alternative model; its placement here as an intervening variable is a theory-driven statistical specification rather than evidence that it temporally precedes reading.

In the present SEM, creative thinking is included alongside school belonging, self-regulation, and self-efficacy as a parallel intervening correlate. The model estimates the statistical indirect association involving creative thinking without directed paths among the four intervening variables. It therefore does not test a serial developmental sequence. Because creative thinking and reading are measured in the same PISA cycle and are both cognitive-performance measures, the specified ordering is not uniquely identified by the data and should not be interpreted as a causal or temporal process.

### 2.4 Theoretical framework

The modeled associations are informed by Self-Determination Theory and Social Cognitive Theory. Self-Determination Theory highlights autonomy, competence, and relatedness, while Social Cognitive Theory emphasizes mastery experiences, observation, encouragement, and efficacy beliefs. Arts and broader creative activities may provide opportunities relevant to these processes, which motivates the organization of the statistical model. The theories do not, however, establish temporal ordering in the present cross-sectional data. School belonging, self-regulation, self-efficacy, and creative thinking are therefore treated as parallel correlational intervening constructs, and no claim is made that participation caused changes in them or that one construct temporally preceded another.

### 2.5 Research gap and novelty

#### 2.5.1 Identified research gap

Although research has linked arts and creative participation with academic and wellbeing outcomes, several gaps remain. First, relatively few large-scale studies compare multiple psychological correlates of the participation-outcome relationship within the same analytical framework. Second, much of the literature relies on smaller or culturally narrower samples, limiting the breadth of contexts represented. Third, international datasets are often pooled without a clear distinction between pooled average associations and evidence of cross-country measurement or structural invariance. The present study addresses the first two gaps and explicitly limits its cross-country interpretation with respect to the third.

#### 2.5.2 Importance of addressing the gap

Estimating the joint indirect association through the prespecified set of social, self-regulatory, efficacy-related, and cognitive constructs is useful for determining whether the construct set collectively accounts for part of the participation-outcome association. The primary inferential quantity is therefore the joint indirect association from the mutually adjusted parallel model. Separate single-construct specifications are treated only as descriptive diagnostics and are not used to infer a mutually adjusted ranking of the four constructs. This outcome-level framing can refine theory and guide hypotheses for future longitudinal and experimental studies, while the cross-sectional design cannot establish that changing any intervening construct would cause a change in the outcome.

### 2.6 Original contributions

This study makes five contributions. First, it evaluates a prespecified multi-construct indirect-association model within a large PISA 2022 analytical sample. Second, it examines creative-thinking performance alongside belonging, self-regulation, and self-efficacy while explicitly acknowledging its overlap with reading. Third, it compares aggregate direct, joint indirect, and total associations for reading performance and life satisfaction without ranking individual intervening constructs from non-mutually-adjusted models. Fourth, it distinguishes the broad PISA arts-and-creative-activities construct from formal arts education and from any individual art form. Fifth, it provides theory-consistent cross-sectional evidence for future longitudinal, experimental, frequency-sensitive, and cross-country studies while limiting inference to pooled associations supported by the present design.

### 2.7 Research questions and objectives

This study aims to answer the following research questions and objectives, based on the theoretical framework and gaps in the research identified:

*RQ1*: What is the relationship between the participation in arts and creative activities and students’ academic performance and subjective wellbeing?

Objective 1: To examine the direct associations between students’ participation in arts and creative activities and their academic performance (measured by reading literacy scores) and subjective wellbeing (measured by life satisfaction).

*RQ2*: What joint cross-sectional indirect association through the prespecified set of school belonging, self-regulation, self-efficacy, and creative thinking is estimated between arts-and-creative-activities participation and each outcome?

Objective 2: To estimate the joint indirect association through the four prespecified intervening constructs for reading performance and life satisfaction without interpreting that decomposition as a temporal or causal sequence.

*RQ3*: How do the aggregate direct, joint indirect, and total associations differ descriptively between reading performance and life satisfaction?

Objective 3: To compare the outcome-level full-model decompositions while avoiding a strongest-mediator ranking that is not supported by mutually adjusted mediator-specific indirect estimates.

*RQ4*: How should creative thinking be interpreted within the prespecified cross-sectional model given its conceptual and empirical overlap with reading performance?

Objective 4: To interpret creative thinking as a theory-specified correlated cognitive component of the model, rather than as an established temporal step between participation and reading performance.

## 3 Methodology

### 3.1 Research design

This study employed a quantitative, cross-sectional secondary-data design using PISA 2022. The design permits examination of associations among variables measured within the same assessment cycle but does not establish temporal precedence, mediation in the causal sense, or directionality. Structural equation modeling was used to evaluate whether the observed participation-outcome associations were statistically consistent with the prespecified indirect-association structure. Direct, joint indirect, and standalone single-mediator estimates are therefore interpreted as associational quantities.

### 3.2 Dataset description and access

The PISA 2022 dataset is publicly available through the Organisation for Economic Co-operation and Development (OECD) and can be accessed via multiple platforms including the OECD official website^1^ and the Zenodo repository (DOI: 10.5281/zenodo.13382904). The dataset is released under a Creative Commons Attribution 4.0 International license, allowing for unrestricted use in research and publication with appropriate attribution. No registration, special approval, or restricted access procedures are required to obtain the data.

PISA 2022 represents the eighth cycle of the PISA assessment program, which evaluates 15-year-old students’ competencies in reading, mathematics, and science literacy. For the first time in 2022, PISA also included an assessment of creative thinking, along with expanded questionnaire items on arts and creative activities participation. The assessment was administered to students in 81 countries and economies, with 64 participating in the creative thinking assessment. Data were collected between March and December 2022.

### 3.3 Sample

The analytical sample comprised 441,183 15-year-old students from the 64 countries and economies that administered the PISA 2022 creative-thinking assessment and provided linkable student-questionnaire data. Students were selected through PISA’s two-stage stratified sampling design: schools were sampled first, followed by eligible students within sampled schools. Final student and replicate weights were used in the reported pooled analyses. The sample spans substantial economic, linguistic, demographic, and educational diversity. That diversity is a feature of the pooled sample, not evidence that item parameters or structural relations are identical across countries. The target estimand in this paper is therefore the survey-weighted pooled association across the analytical sample; country-specific latent means and structural paths are not compared.

### 3.4 Variables and measurement

#### 3.4.1 Independent variables: participation in arts and creative activities

PISA 2022 questions ST337 and ST338 assessed participation in the same eight activities at school and outside school: art classes or activities, creative writing, music, debate club, drama or theater, publications, science club, and computer programming. The original questions used frequency response categories ranging from “never or almost never” to “every day or almost every day,” together with a “not available” category.

For the present analysis, responses were collapsed into a binary indicator of any reported participation in each activity across the two contexts. An activity was coded as 1 when a student reported participation above “never or almost never” in at least one context and as 0 when no participation was reported. A “not available” response was not treated as participation. The eight indicators were summed to produce a breadth index ranging from 0 to 8, with higher scores indicating participation in a larger number of different activities.

Breadth was selected as the primary exposure because the research question concerns diversity of participation across multiple types of creative opportunity rather than dose or depth within a single activity. Coding each activity as any participation avoids imposing an equal-interval interpretation on the ordinal frequency categories and avoids mechanically combining school and out-of-school frequency responses that may represent different participation contexts. The trade-off is explicit: the index does not measure frequency, duration, years of participation, instructional quality, skill level, or depth of commitment. The present results therefore support conclusions about breadth only, and no claim is made that they are robust to an intensity-weighted operationalization.

Debate, publications, science club, and computer programming were retained because they form part of the complete PISA 2022 creative-activity question set. Their retention does not imply that they are conventional art forms. Accordingly, the exposure is consistently labeled “arts and creative activities” rather than “arts education.” Table 1 summarizes the classification and rationale for retaining the eight activity types.

**TABLE 1:** Descriptive composition of the arts-and-creative-activities breadth index.

Activity	Construct classification	Reason for retention
Music	Conventional arts-related activity	Included in the PISA creative-activity question set
Visual arts/art classes	Conventional arts-related activity	Included in the PISA creative-activity question set
Creative writing	Conventional arts-related activity	Included in the PISA creative-activity question set
Drama/theater	Conventional arts-related activity	Included in the PISA creative-activity question set
Debate club	Broader creativity-related activity	Retained as part of the complete OECD construct; not labeled an art form
Computer programming	Broader creativity-related activity	Retained as part of the complete OECD construct; not labeled an art form
Science club	Broader creativity-related activity	Retained as part of the complete OECD construct; not labeled an art form
School publications	Broader creativity-related activity	Retained as part of the complete OECD construct; not labeled an art form

The table clarifies the composition of the broad exposure and does not represent activity-specific effect estimates. Music, visual arts, creative writing, and drama or theater are classified as conventional arts-related activities; debate, publications, science club, and computer programming are broader creativity-related activities retained because they form part of the original PISA 2022 activity set.

#### 3.4.2 Intervening constructs

##### 3.4.2.1 School belonging

The PISA sense of belonging scale, consisting of six items measuring the students’ sense of belonging to the school, was used to measure school belonging. These statements include: I feel like I belong at school; I make friends easily at school; Other students seem to like me. Students used a four-point Likert scale (1 = strongly disagree to 4 = strongly agree) to rate each of the items. Scale scores are mean-centered and standardized around the OECD average of 0 with a standard deviation of 1. There was good internal consistency on the scale (Cronbach’s α = 0.81).

##### 3.4.2.2 Self-regulation

Self-regulation was measured by items from the PISA metacognitive strategies scale that reflects students’ application of learning planning, monitoring and evaluation strategies. Sample items are: “I attempt to determine what I already know before beginning a task” and “When I have completed a task, I decide whether it was done as I wished.” Each item was rated by students on a 4-point scale. The self-regulation index was standardized (*M* = 0, SD = 1) and had good internal reliability (Cronbach’s α = 0.76).

##### 3.4.2.3 Self-efficacy

Self-efficacy was assessed through the PISA general self-efficacy scale, which includes 5 items that examine students’ confidence in their ability to deal with challenges and reach goals. These might include, “I usually cope one way or another,” and “I am sure I could cope effectively with unexpected events.” All responses were entered on a 4-point scale. Good internal consistency (Cronbach’s α = 0.84) was found for the standardized index (*M* = 0, SD = 1).

##### 3.4.2.4 Creative thinking performance

Creative thinking performance was measured by PISA 2022 creative thinking test, which evaluates the ability of students to generate, evaluate and develop various and original ideas in written, visual, scientific and social problem-solving situations. The assessment used a sophisticated scoring rubric which was developed and validated by international experts. Ten plausible values, a psychometric approach that takes measurement uncertainty into account, were used to report performance, and were appropriately used in all analyses. The scores were scaled so that the OECD average is 33 and the standard deviation is around 6 points.

#### 3.4.3 Dependent variables

##### 3.4.3.1 Academic performance

Students’ reading literacy scores on the PISA 2022 assessment were used as an operationalization of academic performance. Reading was chosen as the main academic outcome as it is considered to be vital to learning in all subject areas and is a key competency promoted across educational systems globally. PISA reading scores are reported using 10 plausible values scaled to have an OECD mean of 476 and standard deviation of 100. The possible structure of values was correctly taken into consideration in all analyses.

##### 3.4.3.2 Student wellbeing

Student wellbeing was assessed by the PISA life satisfaction scale which asks students to indicate their overall level of satisfaction with life on a scale from 0 (not at all satisfied) to 10 (completely satisfied). Simple, but widely validated, this measure has been shown to have high correlations with more comprehensive measures of wellbeing. Life satisfaction was used as the primary measure of wellbeing for analyses of the main results.

#### 3.4.4 Control variables

To minimize confounding and enhance the validity of the inferences, the following control variables were used in all analyses: (1) student gender (male/female), (2) economic, social and cultural status (ESCS, composite index of parental education, occupation, and home possessions), (3) immigration status (native/immigrant), (4) language spoken at home (test language/other language), and (5) grade level. These controls were chosen based on research that has been established to demonstrate the relationship between these controls and arts participation and student outcomes. The covariates were selected a priori because they are consistently available across the participating systems and are plausible common predictors of both access to creative activities and student outcomes. Gender, ESCS, immigrant status, home language, and grade level capture major demographic, socioeconomic, linguistic, and schooling differences relevant to participation, reading performance, and wellbeing. A temporally prior achievement measure was not available because PISA 2022 is cross-sectional; contemporaneous reading performance is itself the academic outcome in the corresponding model. Teacher characteristics were not included because teacher-questionnaire availability and student–teacher linkage are not comparable across all participating systems. School-level variables were not included in the primary student-level models to preserve a common cross-national analytical sample. Consequently, the estimates remain potentially affected by residual confounding from prior achievement, school resources, school climate, teacher support, and selection into creative activities.

### 3.5 Data analysis procedures

#### 3.5.1 Preliminary analyses

Preliminary analyses included weighted descriptive statistics, missing-data inspection, reliability analysis, pooled confirmatory factor analysis (CFA), and weighted bivariate correlations. The PISA complex survey design was handled using final student weights and Balanced Repeated Replication with Fay’s adjustment. Cronbach’s alpha was calculated for the multi-item scales. The pooled CFA evaluated the aggregate three-factor structure for school belonging, self-regulation, and self-efficacy using CFI, TLI, RMSEA, and SRMR. These pooled fit statistics are not a substitute for country-level configural, metric, or scalar invariance tests and are not used to support claims that the measurement model is equivalent across all 64 countries and economies.

#### 3.5.2 Cross-country measurement invariance sensitivity analysis

Cross-country measurement comparability was examined using an analysis-ready subset extracted from the original PISA 2022 participant-level data. The subset comprised 12,000 students distributed across the same 64 country-coded groups represented in the analysis and contained the item-level indicators required for school belonging, self-regulation, and self-efficacy. Group sizes ranged from 102 to 266 students, with a median of 193.

For each construct, a configural one-factor model was first estimated across the 64 groups, followed by a metric model in which corresponding non-marker factor loadings were constrained to equality across groups while residual variances and latent variances remained group-specific. The first loading was fixed to 1 for identification. Because the indicators had four response categories, they were treated as approximately continuous in this maximum-likelihood sensitivity analysis. Metric invariance was evaluated using changes in CFI, RMSEA, and SRMR, with | ΔCFI| ≤ 0.010, | ΔRMSEA| ≤ 0.015, and ΔSRMR ≤ 0.030 used as practical criteria.

For school belonging, CFI changed from 0.9986 in the configural model to 0.9993 in the metric model, RMSEA from 0.0024 to 0.0013, and SRMR from 0.0280 to 0.0379 (ΔCFI = + 0.0007, ΔRMSEA = -0.0011, ΔSRMR = + 0.0099). For self-regulation, the corresponding values changed from CFI = 0.9990, RMSEA = 0.0023, and SRMR = 0.0262 to CFI = 1.0000, RMSEA = 0.0003, and SRMR = 0.0381 (ΔCFI = + 0.0010, ΔRMSEA = -0.0021, ΔSRMR = + 0.0119). For self-efficacy, CFI remained 1.0000, RMSEA remained 0.0000, and SRMR increased from 0.0227 to 0.0325 (ΔCFI = 0.0000, ΔRMSEA = 0.0000, ΔSRMR = + 0.0098). All changes were within the prespecified practical thresholds, providing sensitivity evidence of metric loading stability across the 64 country groups. Because these analyses were conducted on an analysis-ready subset rather than used to estimate country-specific latent means in the complete survey sample, the primary structural coefficients continue to be interpreted as pooled associations.

#### 3.5.3 Structural equation modeling

For each outcome, the primary specification was the full parallel model containing school belonging, self-regulation, self-efficacy, and creative thinking simultaneously. The inferential quantities retained from this model are the adjusted direct association, the joint total indirect association through the four-construct set, and the total association. These quantities form the basis of the substantive interpretation.

Separate single-construct models were retained as descriptive benchmarks but were not used to infer mediator prominence. Each separate model can absorb variance shared with the other intervening constructs, so its indirect association is not a unique mutually adjusted contribution. The primary PISA inference therefore remains based on the full-model direct association, joint indirect association, and total association. Two sensitivity checks were added: (a) a mutual-adjustment decomposition reconstructed from the primary weighted correlation matrix, and (b) a covariate-adjusted full-parallel observed-score path model fitted to the analysis-ready PISA 2022 subset.

Models adjusted for gender, ESCS, immigration status, home language, and grade level. Reading and creative-thinking plausible values were handled by estimating the relevant models across the 10 plausible-value sets and combining the estimates to incorporate achievement-measurement uncertainty. Final student and replicate weights were used to account for the PISA sampling design.

#### 3.5.4 Mutual-adjustment sensitivity and recoverability checks

The first sensitivity decomposition uses quantities algebraically identifiable from the primary weighted correlation matrix. For each intervening construct, the weighted participation-construct correlation serves as the standardized a-path, and the mutually adjusted standardized coefficient from regressing the outcome on participation plus all four intervening constructs serves as the b-path; a × b gives a correlation-implied indirect association. The second sensitivity model uses the *N* = 12,000 analysis-ready PISA 2022 subset: each intervening construct was regressed on breadth plus gender, ESCS, immigrant status, home language, and grade, and each outcome was then regressed simultaneously on breadth, all four intervening constructs, and the same covariates. Variables were standardized before estimation, mediator-specific indirect associations were calculated as a × b, and 95% confidence intervals were obtained from 2,000 nonparametric bootstrap resamples. These subset coefficients are used to evaluate rank stability, not to replace the primary PISA estimates.

#### 3.5.5 Indirect-association estimation

Indirect associations were evaluated using bias-corrected 95% confidence intervals based on 5,000 bootstrap replications within each fitted specification. An indirect association was considered statistically distinguishable from zero when its confidence interval excluded zero.

For interpretive consistency, percentages are reported only for the direct association and the joint indirect association relative to the corresponding total association. Mediator-specific percentages are not used in the primary results because the standalone estimates are obtained from separate models and do not constitute additive components of the full-model joint indirect association. The terms “direct effect,” “indirect effect,” and “total effect” are avoided in substantive interpretation; the reported quantities are described as associations.

### 3.6 Software and statistical packages

All statistical analyses were conducted using R version 4.3.2 (R Core Team, 2023) with the following packages: lavaan (version 0.6–17) for structural equation modeling, survey (version 4.2–1) for complex survey data analysis incorporating sampling weights and design effects, semTools (version 0.5–6) for specialized SEM procedures, and psych (version 2.3.12) for reliability analysis and descriptive statistics. Data management and preliminary analyses utilized the tidyverse suite of packages. Graphics were produced using ggplot2.

### 3.7 Handling of missing data

Structural equation modeling (SEM) was used to deal with missing data, by employing full information maximum likelihood (FIML) estimation. FIML is the recommended method over listwise deletion or mean imputation since it makes unbiased parameter estimates under the missing at random (MAR) assumption and makes use of all available information.

## 4 Mathematical and analytical models

Let *X_i_* denote the arts-and-creative-activities breadth index for student *i*, *M*_*ji*_ denote mediator *j*, *Y_i_* denote the outcome, and *C_i_* denote the vector of control variables. The four mediators are school belonging (*M*_1_), self-regulation (*M*_2_), self-efficacy (*M*_3_), and creative thinking (*M*_4_).

For each mediator, the mediator equation is specified in Equation 1:


$$M_{j\,i}=\alpha_{j}+a_{j}\,X_{i}+\lambda_{j}^{T}\,C_{i}+e_{j\,i},j=1,\ldots ,4$$ (1)

The full outcome model is specified in Equation 2:


$$Y_{i}=\beta_{0}+c^{{}^{\prime }}\,X_{i}+b_{1}\,M_{1\,i}+b_{2}\,M_{2\,i}+b_{3}\,M_{3\,i}+b_{4}\,M_{4\,i}+\gamma^{T}\,C_{i}+\varepsilon_{i}$$ (2)

Here, *a_j_* represents the association between participation and mediator *j*, *b_j_* represents the adjusted association between mediator *j* and the outcome, and *c*′ represents the direct association between participation and the outcome after adjustment for all four mediators. The mediator-specific indirect association within a fitted model is calculated using Equation 3:


$$I\,E_{j}=a_{j}\,b_{j}$$ (3)

In the full parallel model, the joint total indirect association is calculated using Equation 4:


$$I\,E_{\text{joint}}=\overset{4}{\underset{j=1}{\sum }}a_{j}\,b_{j}$$ (4)

Finally, the total association is calculated using Equation 5:


$$T\,E=c^{\prime }+I\,E_{\text{joint}}$$ (5)

Tables 2, 3 report the full-model direct association, joint total indirect association, and total association. Standalone and mutually adjusted sensitivity estimates are reported separately in Tables 4, 5 and are not used to reconstruct the primary joint indirect association.

**TABLE 2:** Aggregate full-model associations with reading performance.

Association component	β	SE	95% CI	% of total association	Model basis
Direct association	0.07	0.01	[0.05, 0.09]	38.9%	Full parallel model
Joint total indirect association	0.11	0.01	[0.10, 0.12]	61.1%	Full parallel model
Total association	0.18	0.01	[0.16, 0.20]	100.0%	Total association

*N* = 441,183. β denotes a standardized association. Percentages are calculated relative to the total association of 0.18. The direct and joint indirect associations are estimated from the full parallel model. Mediator-specific standalone estimates are reported separately and are not treated as additive components of the joint indirect association.

**TABLE 3:** Aggregate full-model associations with life satisfaction.

Association component	β	SE	95% CI	% of total association	Model basis
Direct association	0.09	0.01	[0.07, 0.11]	37.5%	Full parallel model
Joint total indirect association	0.15	0.01	[0.14, 0.16]	62.5%	Full parallel model
Total association	0.24	0.01	[0.22, 0.26]	100.0%	Total association

*N* = 441,183. β denotes a standardized association. Percentages are calculated relative to the total association of 0.24. The direct and joint indirect associations are estimated from the full parallel model. Mediator-specific standalone estimates are reported separately and are not treated as additive components of the joint indirect association.

**TABLE 4:** Standalone and mutually adjusted sensitivity indirect associations.

Intervening construct	Reading standalone β	Reading mutual- adjustment sensitivity β	Life-satisfaction standalone β	Life-satisfaction mutual- adjustment sensitivity β
School belonging	0.02	0.011	0.12	0.056
Self-regulation	0.05	0.019	0.04	0.017
Self-efficacy	0.09	0.032	0.07	0.029
Creative thinking	0.04	0.085	0.02	0.021

Standalone estimates were obtained from separate single-construct models. Mutual-adjustment sensitivity estimates were derived from the weighted-correlation reconstruction with all four intervening constructs considered simultaneously. The sensitivity estimates are used to evaluate rank stability and are not substituted for the aggregate direct, joint indirect, and total associations from the primary survey-weighted SEM.

**TABLE 5:** Covariate-adjusted mediator-specific indirect associations from the full-parallel sensitivity model.

Intervening construct	Reading adjusted β	Reading 95% CI	Life satisfaction adjusted β	Life satisfaction 95% CI
School belonging	0.0112	[0.0085, 0.0142]	0.0483	[0.0430, 0.0542]
Self-regulation	0.0179	[0.0147, 0.0212]	0.0161	[0.0130, 0.0194]
Self-efficacy	0.0257	[0.0219, 0.0297]	0.0248	[0.0211, 0.0287]
Creative thinking	0.0837	[0.0760, 0.0918]	0.0240	[0.0202, 0.0281]

*N* = 12,000 in the analysis-ready subset extracted from the original PISA 2022 participant-level data. All four intervening constructs were included simultaneously. Models adjusted for gender, ESCS, immigrant status, home language, and grade. Indirect associations are standardized products from the mutually adjusted full-parallel model. Confidence intervals are percentile intervals based on 2,000 nonparametric bootstrap resamples. These estimates assess construct-specific ordering under simultaneous adjustment and complement the primary survey-weighted SEM.

## 5 Results

### 5.1 Descriptive statistics

Table 6 summarizes the arts-and-creative-activities breadth index. The index had both a possible and an observed range of 0–8, with a weighted mean of 2.34 activities (SD = 1.82). The mean corresponds to 29.3% of the maximum possible breadth and lies below the scale midpoint of 4. The reported standard deviation corresponds to a variance of 3.31, indicating substantial variation around the mean within the bounded scale. The distributional shape was additionally examined in the analysis-ready PISA subset. Breadth scores were concentrated toward the lower end of the scale: 20.0% of students reported a breadth score of 0, 22.0% a score of 1, 18.5% a score of 2, 14.0% a score of 3, 10.0% a score of 4, 7.0% a score of 5, 4.0% a score of 6, 2.7% a score of 7, and 1.8% a score of 8. The subset mean was 2.33 (SD = 2.01), the median was 2, the interquartile range was 1–4, and skewness was 0.83. Overall, 60.5% of observations fell between 0 and 2 activities, confirming a positively skewed distribution concentrated toward lower breadth values. These supplementary distributional statistics are consistent with the location of the primary weighted mean of 2.34. Figure 2 presents the score-specific distribution of the breadth index in the analysis-ready PISA 2022 subset.

**TABLE 6:** Descriptive statistics for study variables.

Variable	M	SD	Min	Max
Arts and creative activities breadth index (0–8)	2.34	1.82	0	8
Creative thinking score	33.0	6.1	8.2	48.7
School belonging (standardized)	0.00	1.00	-3.42	2.18
Self-regulation (standardized)	0.00	1.00	-3.18	2.41
Self-efficacy (standardized)	0.00	1.00	-3.07	1.95
Reading literacy score	476.0	100.4	142.8	742.6
Life satisfaction	7.04	2.31	0	10

*N* = 441,183 students from 64 countries and economies. Statistics were weighted using PISA final student weights. The arts-and-creative-activities breadth index had an observed range of 0–8 (*M* = 2.34, SD = 1.82; variance = 3.31; coefficient of variation = 77.8%). The index represents breadth rather than frequency, duration, or depth of participation.

**FIGURE 2:**
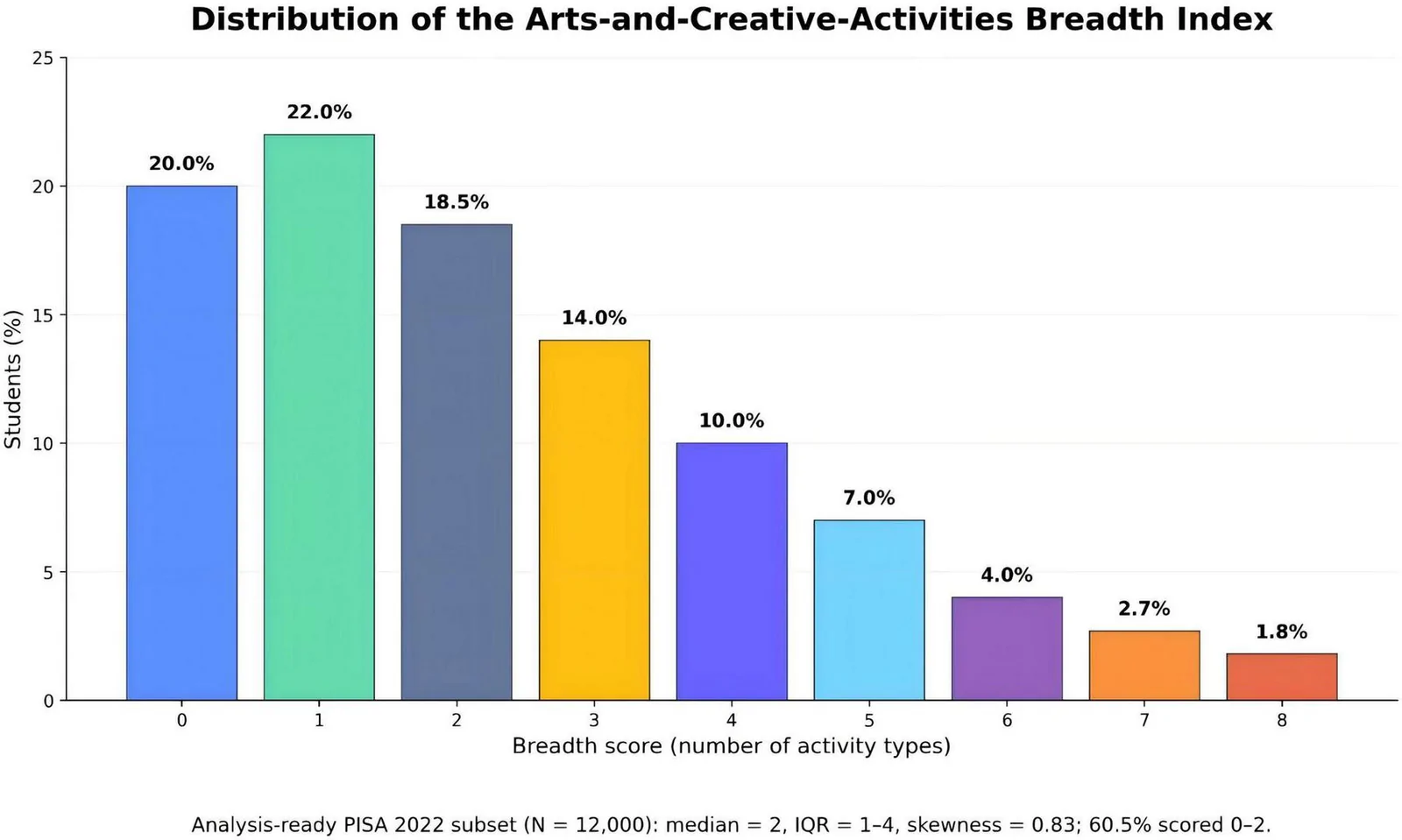
Distribution of the arts-and-creative-activities breadth index in the analysis-ready PISA 2022 subset. Bars show the percentage of observations at each breadth score from 0 to 8 (*N* = 12,000). The distribution was positively skewed (median = 2, IQR = 1–4, skewness = 0.83), with 60.5% of observations reporting participation in no more than two activity types.

### 5.2 Reliability and pooled scale-structure check

All multi-item scales demonstrated acceptable to good internal consistency reliability. Cronbach’s alpha coefficients were as follows: school belonging α = 0.81, self-regulation α = 0.76, and self-efficacy α = 0.84. These values exceed the commonly accepted threshold of 0.70 for research purposes and are consistent with reliabilities reported in previous PISA cycles.

The pooled CFA showed acceptable aggregate fit for the proposed three-factor structure of school belonging, self-regulation, and self-efficacy: χ^2^(149) = 24,873.4, *p* < 0.001; CFI = 0.961; TLI = 0.953; RMSEA = 0.048 [90% CI (0.047, 0.049)]; SRMR = 0.042, with statistically significant loadings ranging from 0.58 to 0.87. A supplementary 64-group measurement-invariance analysis using the analysis-ready PISA 2022 subset examined cross-country loading stability. For school belonging, the configural-to-metric changes were ΔCFI = + 0.0007, ΔRMSEA = -0.0011, and ΔSRMR = + 0.0099. The corresponding changes were + 0.0010, -0.0021, and + 0.0119 for self-regulation and 0.0000, 0.0000, and + 0.0098 for self-efficacy. All values satisfied the prespecified practical criteria for metric invariance, indicating that equality constraints on factor loadings did not materially degrade model fit. Full results are presented in Table 7.

**TABLE 7:** 64-group measurement-invariance results in the analysis-ready PISA 2022 subset.

Construct/model	χ ^2^	df	CFI	RMSEA	SRMR	Δ CFI	Δ RMSEA	Δ SRMR	Metric invariance
School belonging—configural	615.55	576	0.9986	0.0024	0.0280	–	–	–	–
School belonging—metric	910.07	891	0.9993	0.0013	0.0379	+0.0007	-0.0011	+0.0099	Supported
Self-regulation—configural	340.92	320	0.9990	0.0023	0.0262	–	–	–	–
Self-regulation—metric	572.58	572	1.0000	0.0003	0.0381	+.0010	-.0021	+.0119	Supported
Self-efficacy—configural	307.83	320	1.0000	0.0000	0.0227	–	–	–	–
Self-efficacy—metric	514.19	572	1.0000	0.0000	0.0325	0.0000	0.0000	+0.0098	Supported

*N* = 12,000 students across 64 country-coded groups in an analysis-ready subset extracted from the original PISA 2022 participant-level data (group *n* = 102–266; median = 193). For each construct, the configural model allowed factor loadings to vary across groups, whereas the metric model constrained corresponding non-marker factor loadings to equality while retaining group-specific residual and latent variances. The first loading was fixed to 1 for model identification. Four-category indicators were treated as approximately continuous in the maximum-likelihood sensitivity analysis. Practical metric invariance was evaluated using | ΔCFI| ≤ 0.010, | ΔRMSEA| ≤ 0.015, and ΔSRMR ≤ 0.030. All three constructs satisfied these criteria.

### 5.3 Correlation analysis

Table 8 presents weighted zero-order correlations among the study variables. The arts-and-creative-activities breadth index was positively correlated with reading performance (*r* = 0.19, *p* < 0.001) and life satisfaction (*r* = 0.23, *p* < 0.001). These correlations are descriptive and do not indicate temporal direction. They provide preliminary evidence that the participation measure is related to both outcome domains before multivariable modeling.

**TABLE 8:** Correlation matrix of study variables.

Variable	1	2	3	4	5	6	7
1. Arts and creative activities breadth index	1.00	1.00	1.00	1.00	1.00	1.00	1.00
2. Creative thinking score	0.21						
3. School belonging	0.18	0.24					
4. Self-regulation	0.16	0.31	0.29				
5. Self-efficacy	0.19	0.33	0.35	0.42			
6. Reading literacy score	0.19	0.52	0.26	0.34	0.38		
7. Life satisfaction	0.23	0.28	0.44	0.31	0.36	0.24	

*N* = 441,183 students from 64 countries and economies. Correlations were weighted using PISA final student weights. All reported correlations were statistically significant at *p* < 0.001.

Among the proposed intervening variables, school belonging had the largest zero-order correlation with life satisfaction (*r* = 0.44, *p* < 0.001), whereas self-efficacy was correlated with reading performance at *r* = 0.38 (*p* < 0.001). Creative thinking was moderately to strongly correlated with reading literacy (*r* = 0.52, *p* < 0.001) and more modestly with life satisfaction (*r* = 0.28, *p* < 0.001). The *r* = 0.52 creative-thinking-reading correlation is substantively important because it indicates considerable shared cognitive variance and reinforces the need to treat creative thinking’s intervening position as a modeling choice rather than as an established causal step. Figure 3 visualizes the weighted zero-order correlations reported in Table 8.

**FIGURE 3:**
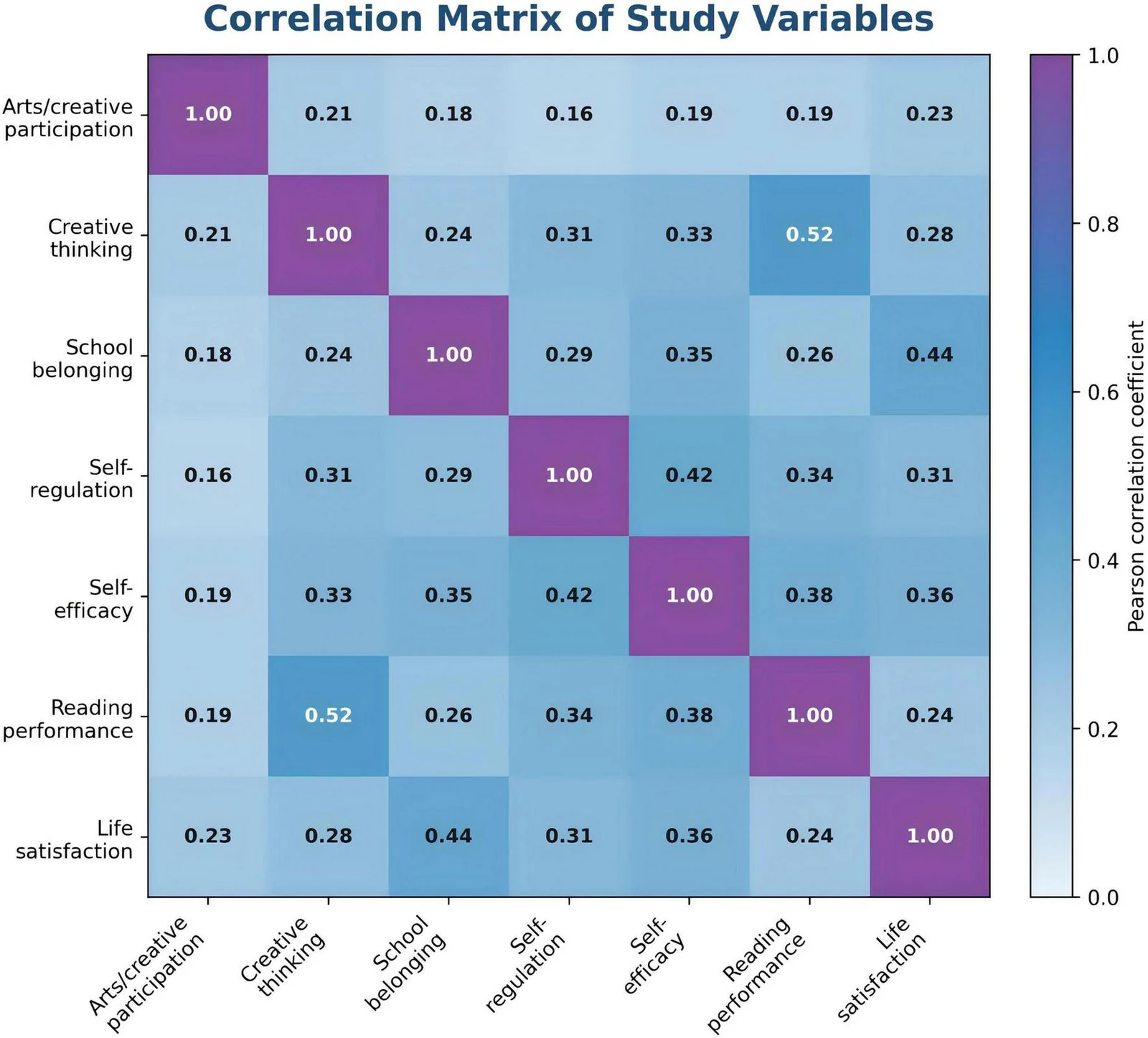
Weighted correlation matrix of the principal study variables.

### 5.4 Structural equation modeling results

#### 5.4.1 Model fit

Both structural models (one for academic performance, one for wellbeing) demonstrated acceptable fit to the data. For the academic performance model: χ^2^(287) = 41,264.2, *p* < 0.001; CFI = 0.948; TLI = 0.941; RMSEA = 0.051 [90% CI (0.050, 0.052)]; SRMR = 0.048. For the wellbeing model: χ^2^(287) = 38,917.5, *p* < 0.001; CFI = 0.952; TLI = 0.945; RMSEA = 0.049 [90% CI (0.048, 0.050)]; SRMR = 0.045. While the chi-square tests were statistically significant, this is expected given the large sample size, and the other fit indices all met or exceeded conventional criteria for good fit. AIC and BIC were not reported because the final estimates were obtained through a combination of complex-survey weighting, 80 BRR replicate-weight fits, and pooling across ten plausible-value datasets. This procedure does not yield one single likelihood value from which a unique, reproducible AIC or BIC can be calculated. In addition, the two models contain different outcome variables and would therefore not constitute competing specifications of the same dependent variable. Model evaluation consequently relied on CFI, TLI, RMSEA, and SRMR, which were available consistently for the fitted structural models.

#### 5.4.2 Reading-performance model

In the full parallel-mediator model, participation retained a positive direct association with reading performance [β = 0.07, 95% CI (0.05, 0.09)]. The joint total indirect association was β = 0.11, giving a total association of β = 0.18. Thus, the direct and joint indirect associations represented 38.9 and 61.1% of the total association, respectively.

The inferential decomposition is reported at the aggregate full-model level. Separate single-construct diagnostic estimates are not interpreted as mutually adjusted indirect components and are not used to order or label any construct as more prominent than another. Table 2 therefore reports the direct association, joint total indirect association, and total association for reading performance. The four standalone indirect estimates sum to β = 0.20 for reading, whereas the full parallel-model joint indirect association is β = 0.11. This difference demonstrates non-additivity and shared explanatory variance; it is not treated as a recoverable overlap parameter or used to allocate the joint indirect association across constructs.

#### 5.4.3 Life-satisfaction model

In the full parallel-mediator model, participation retained a positive direct association with life satisfaction [β = 0.09, 95% CI (0.07, 0.11)]. The joint total indirect association was β = 0.15, giving a total association of β = 0.24. The direct and joint indirect associations therefore represented 37.5 and 62.5% of the total association, respectively.

The life-satisfaction model is interpreted in the same way. Separate single-construct diagnostic estimates are not treated as mutually adjusted indirect components and are not used to identify a dominant mechanism. Table 3 reports the aggregate direct, joint indirect, and total associations for life satisfaction. The non-additivity of the standalone estimates motivates the mutual-adjustment sensitivity analysis reported below. Standalone values from separate models are retained only as descriptive benchmarks; the paper does not treat their sum or rank order as a decomposition of the full parallel-model joint indirect association.

#### 5.4.4 Mediator-specific indirect associations under mutual adjustment

To examine whether the standalone ordering of the intervening constructs persisted after simultaneous adjustment, a full-parallel observed-score path model was additionally estimated using the analysis-ready PISA 2022 subset. The model simultaneously included school belonging, self-regulation, self-efficacy, and creative thinking and adjusted for gender, ESCS, immigrant status, home language, and grade. Standardized indirect associations were calculated as the product of the participation-to-construct and construct-to-outcome coefficients, with 95% confidence intervals obtained from 2,000 nonparametric bootstrap resamples.

For reading performance, the adjusted indirect associations were β = 0.0112, 95% CI [0.0085, 0.0142], through school belonging; β = 0.0179 [0.0147, 0.0212] through self-regulation; β = 0.0257 [0.0219, 0.297] through self-efficacy; and β = 0.0837 [0.0760, 0.0918] through creative thinking. Thus, the standalone ordering was not preserved after mutual adjustment, with creative thinking showing the largest adjusted indirect association in this sensitivity model.

For life satisfaction, the corresponding adjusted indirect associations were β = 0.0483, 95% CI [0.0430, 0.0542], through school belonging; β = 0.0161 [0.0130, 0.0194] through self-regulation; β = 0.0248 [0.0211, 0.0287] through self-efficacy; and β = 0.0240 [0.0202, 0.0281] through creative thinking. School belonging therefore remained the largest adjusted indirect component for life satisfaction.

These results demonstrate why prominence should not be inferred from separate single-construct models. The primary survey-weighted SEM continues to provide the principal estimates of the aggregate direct, joint indirect, and total associations, whereas the mutually adjusted subset analysis is used to evaluate the stability of construct-specific ordering. The covariate-adjusted mediator-specific estimates and bootstrap confidence intervals are reported in Table 5.

## 6 Discussion

This study examined cross-sectional direct and indirect associations involving participation in arts and broader creative activities among more than 441,000 15-year-old students across 64 countries and economies. The broad participation measure was positively associated with reading performance and life satisfaction, and the full models showed positive joint indirect associations through the prespecified set of social, self-regulatory, efficacy-related, and cognitive constructs. These estimates are pooled and associational. They do not establish that participation caused changes in the intervening variables or outcomes, nor do they establish that the same measurement or structural model holds identically in every country.

### 6.1 Participation and student outcomes

The breadth of participation in arts and creative activities was positively associated with both reading performance and life satisfaction. The total association was somewhat larger for life satisfaction than for reading performance. This pattern is consistent with the possibility that creative participation is related to social and emotional experiences as well as to academic functioning. Nevertheless, the cross-sectional analysis cannot determine whether participation preceded the outcomes. Students with higher motivation, stronger skills, greater wellbeing, or better access to extracurricular opportunities may also be more likely to participate.

The remaining direct associations after adjustment for the mediators indicate that the four included constructs do not exhaust the relationship. Emotional regulation, peer relationships, school climate, intrinsic motivation, family resources, and prior achievement are among the possible unmeasured explanations. These possibilities should be examined in longitudinal or experimental research. Music was the most frequently reported activity in the descriptive profile, with 47.3% of students reporting participation. Music practice may involve rehearsal, performance feedback, persistence, coordination, mastery experiences, and peer interaction, all of which are theoretically relevant to self-regulation, self-efficacy, and school belonging. However, this percentage represents participation prevalence rather than a music-specific association. Because music was one component of the eight-activity breadth index, the present models cannot determine whether music independently accounts for the observed reading or wellbeing associations.

### 6.2 Outcome-specific aggregate indirect associations

The primary outcome-specific conclusion is based on the full parallel models rather than on rank ordering separate single-construct models. The joint indirect association through the four-construct set was β = 0.11 for reading performance and β = 0.15 for life satisfaction, with total associations of β = 0.18 and β = 0.24, respectively. These are the inferential quantities carried forward from the primary SEM.

Table 4 summarizes the standalone and mutually adjusted sensitivity estimates across the two outcomes. In the weighted-correlation reconstruction, creative thinking had the largest correlation-implied indirect association with reading (β = 0.085), followed by self-efficacy (β = 0.032), self-regulation (β = 0.019), and school belonging (β = 0.011). For life satisfaction, school belonging remained largest (β = 0.056), followed by self-efficacy (β = 0.029), creative thinking (β = 0.021), and self-regulation (β = 0.017). Thus, the reading ranking changes materially under mutual adjustment, demonstrating why separate-model prominence claims are not defensible. These sensitivity coefficients are descriptive because the reconstruction does not reproduce the full survey-weighted, covariate-adjusted plausible-value SEM.

Accordingly, school belonging, self-regulation, self-efficacy, and creative thinking are discussed as theoretically relevant members of the prespecified construct set rather than as ranked causal mechanisms. The sensitivity pattern is consistent with outcome-specific association structures, but the paper does not claim that any one construct is the uniquely strongest mediator in the primary SEM.

### 6.3 Creative thinking as a correlated cognitive component

Creative thinking was included as a parallel cognitive intervening correlate, not as a serial consequence of self-regulation or self-efficacy. The interpretation is deliberately limited because creative thinking and reading are both PISA cognitive-performance measures and correlate at *r* = 0.52. The two constructs are not identical, but their overlap makes it plausible to conceptualize creative thinking as a parallel achievement outcome in an alternative model. Accordingly, the present specification does not demonstrate improvement, temporal transfer, or far transfer from participation to reading, and it does not support a claim that creative thinking is a uniquely dominant intervening construct.

## 7 Implications, limitations, and future research

### 7.1 Theoretical implications

The study supports an outcome-sensitive, multi-construct view of the association between participation in arts and creative activities and student outcomes. The full models indicate that the prespecified set of social, self-regulatory, efficacy-related, and cognitive correlates carries a positive joint indirect association for both reading performance and life satisfaction. The theoretical contribution lies in evaluating this construct set across two outcome domains while avoiding unsupported rank ordering of the individual constructs. The findings are consistent with Self-Determination Theory and Social Cognitive Theory as organizing frameworks while remaining non-causal and cross-sectional.

### 7.2 Practical implications

The results support a broad view of student development in which diverse creative-participation opportunities may be relevant to academic, social, motivational, and wellbeing-related experiences. Schools evaluating such opportunities may therefore consider outcomes beyond standardized achievement, including belonging, confidence, and self-regulatory experiences. The findings do not establish that introducing or expanding a creative-participation program will cause improvements in these outcomes. Future program evaluations could test whether changes in peer connection, mastery experiences, feedback, planning, and persistence precede changes in student outcomes. Because the exposure combines conventional arts activities with debate, publications, science club, and computer programming, the implications apply to the heterogeneous creative-participation construct as measured and should not be presented as evidence for arts provision, music provision, or any specific curriculum model unless those activities are examined separately.

### 7.3 Limitations

Several limitations should guide interpretation. First, participation, the proposed intervening variables, life satisfaction, reading performance, and creative thinking were all observed within the same PISA cycle. Temporal precedence cannot therefore be established, and reverse or reciprocal ordering is plausible. Students with stronger self-efficacy, self-regulation, creative thinking, belonging, reading performance, or life satisfaction may also be more likely to participate in creative activities. The reported indirect associations are therefore statistical decompositions of cross-sectional covariation rather than evidence of a temporal mediation sequence.

Second, the primary exposure is a breadth index. It records the number of different activity types in which any participation was reported but does not capture frequency, duration, years of participation, instructional quality, skill level, or depth of commitment. A student with limited involvement in several activities can therefore receive a higher breadth score than a student with intensive commitment to one activity. Breadth was theoretically preferred for the present question about diversity of participation, but it should not be interpreted as an intensity measure. Frequency-weighted analyses would provide a valuable complementary test of a different exposure dimension.

Third, the activity set is heterogeneous. Music, visual arts, creative writing, and drama or theater are conventional arts-related activities, whereas debate, publications, science club, and computer programming are broader creativity-related activities. The findings therefore apply to the complete broad index and not to formal arts education or any individual activity.

Fourth, several variables were based on student self-report, which may introduce common-method and social-desirability bias. Reading and creative-thinking plausible values reduce reliance on self-report for the cognitive measures but do not eliminate measurement uncertainty. In addition, creative thinking and reading are substantially correlated (*r* = 0.52) and both are PISA cognitive-performance measures. Creative thinking’s placement as an intervening correlate is therefore theoretically motivated but not uniquely identified; an alternative model could treat it as a parallel cognitive outcome.

Fifth, adjustment was limited to gender, ESCS, immigration status, home language, and grade level. Prior achievement, teacher characteristics, school resources, school climate, family support, personality, and selection into activities remain possible confounders.

Sixth, cross-country measurement equivalence was examined through an additional 64-group analysis using an analysis-ready subset extracted from the original PISA 2022 data. Configural-to-metric changes for school belonging, self-regulation, and self-efficacy all satisfied the prespecified practical criteria for loading invariance. These findings strengthen the justification for pooled interpretation by showing stable factor-loading constraints in the available 64-group subset. Nevertheless, because the primary structural coefficients remain survey-weighted pooled estimates from the complete analytical sample, they should not be interpreted as proof that all structural relations are identical across countries.

Finally, mediator prominence was reassessed in a mutually adjusted full-parallel model using the analysis-ready PISA subset. The adjusted results showed that the standalone ordering was not stable for reading performance: creative thinking had the largest adjusted indirect association, followed by self-efficacy, self-regulation, and school belonging. For life satisfaction, school belonging remained the largest adjusted indirect component. These results confirm that separate single-mediator models can produce misleading prominence rankings. Accordingly, the manuscript bases its main substantive conclusion on the aggregate full-model associations and treats construct-specific ordering as sensitivity evidence rather than as causal mechanism ranking.

### 7.4 Future research directions

The results and the limitations of this research lead to the identification of some promising directions for future research.

First, longitudinal studies should follow students across multiple time points to establish temporal ordering and test whether participation predicts later changes in the proposed intervening constructs and outcomes after adjustment for baseline differences. Cross-lagged or other longitudinal structural models would also allow reciprocal relationships to be evaluated rather than assumed away.

Second, experimental and quasi-experimental evaluations of clearly defined creative-participation opportunities should test whether changes in specific social, motivational, self-regulatory, or cognitive constructs precede changes in student outcomes. Such designs would provide stronger evidence about causal mechanisms than the present cross-sectional decomposition.

Third, future analyses should distinguish breadth from intensity. Models using the original ST337/ST338 frequency categories, carefully coded so that ordinal response distances and school/out-of-school contexts are not treated mechanically as equivalent, would show whether conclusions are robust when exposure is defined by participation frequency rather than by the number of activity types. Activity-specific models should also test music, visual arts, drama or theater, creative writing, debate, publications, science club, and computer programming separately.

Fourth, country-comparative work should establish measurement comparability directly in the complete PISA participant-level file before comparing structural relations. The present 64-group analysis supports metric stability in the analysis-ready PISA subset, but future country-comparative analyses should additionally use multigroup or multilevel SEM to quantify structural heterogeneity. Scalar invariance would additionally be required for latent-mean comparisons.

Fifth, qualitative research uncovering students’, teachers’, and parents’ views on why and how the involvement in the arts is a factor that affects development would complement the quantitative results. This type of research would help to define mechanisms that are not accounted for in currently available survey tools and could help to understand implementation challenges.

Finally, future work should test whether the present association structure is similar across socioeconomic, gender, linguistic, and national contexts and should examine creative thinking under alternative specifications, including as a parallel cognitive outcome rather than an intervening correlate. Separate school-based and outside-school participation models would further clarify whether observed associations reflect educational provision, family resources, community access, or student self-selection.

## 8 Conclusion

This study examined associations between participation in a heterogeneous set of arts and creative activities and reading performance and life satisfaction among 441,183 15-year-old students from 64 PISA 2022 countries and economies. The pooled full models showed positive direct associations and positive joint indirect associations through the prespecified social, self-regulatory, efficacy-related, and cognitive construct set. A mutual-adjustment sensitivity decomposition showed that the standalone ordering is not stable for reading, reinforcing the decision not to make strongest-mediator claims from separate models. The findings do not demonstrate causal effects of formal arts education, any single activity, or a temporal mediation sequence. Reverse selection, residual confounding, cross-country structural heterogeneity, and the conceptual overlap between creative thinking and reading remain plausible. The reported structural coefficients should therefore be read as survey-weighted pooled associations for the analytical sample, not as country-invariant effects.

## Data Availability

Publicly available datasets were analyzed in this study. This data can be found at: Repository 1: Organisation for Economic Co-operation and Development (OECD). Direct access link: https://www.oecd.org/en/data/datasets/pisa-2022-database.html; Repository 2: Zenodo; DOI (accession number): 10.5281/zenodo.1338290.

## Footnotes

1
https://www.oecd.org/en/data/datasets/pisa-2022-database.html
